# A new formulation of Bacillus thuringiensis: UV protection and sustained release mosquito larvae studies

**DOI:** 10.1038/srep39425

**Published:** 2016-12-22

**Authors:** Lingling Zhang, Xiaojuan Zhang, Yi Zhang, Songqin Wu, Ivan Gelbič, Lei Xu, Xiong Guan

**Affiliations:** 1 State Key Laboratory of Ecological Pest Control for Fujian and Taiwan Crops, School of Life Sciences Fujian Agriculture and Forestry University, 350002, Fuzhou, Fujian, People’s Republic of China; 2 Fujian-Taiwan Joint Center for Ecological Control of Crop Pests, Fuzhou, 350002, Fuzhou, Fujian, People’s Republic of China; 3 Biology Centre of the Czech Academy of Science, Institute of Entomology, Branišovská 31, 37005, České, Budějovice, Czech Republic

## Abstract

Persistence of *Bacillus thuringiensis* is an important factor in determining the success of this product as a pest control agent. In this report we present the development of a highly active mosquitocidal formulation with high resistance to UV. LLP29-M19 strain of Bt, selected by repeated exposure to UV was found to be highly resistant to UV. The product was optimized and the methods used were statistically analyzed. Using single-factor experiments it was determined that the optimal concentration of sodium alginate, CaCl_2_ and hollow glass beads in the formulation were 1.0%, 2.0% and 3.5%, respectively. Plackett-Burman design was used to screen the interaction of the three factors, CaCl_2_, sodium alginate and hollow glass beads in the sustained-release formulation. The best combined concentration and mutual effects of the three factors were optimized by response surface methodology. The results showed that the most favorable composition was sodium alginate 0.78%, CaCl_2_ 4.52%, hollow glass bead 3.12%, bacterial powder 3.0%, melanin 0.015%, sodium benzoate 0.2%, and mouse feed 0.5%, resulting in the immobilization time of 4.5 h, at which time the corrected sustained-release virulence rose 2391.67 fold, which was 6.07-fold higher than the basic formulation and deviated only 5.0% from the value predicted by RSM.

*Bacillus thuringiensis* (Bt) is a spore-forming gram-positive bacterium, which produces insecticidal crystal proteins (ICPs) that is toxic to more than 3,000 different insects and other pests belonging to Lepidoptera, Diptera, Coleoptera, Hemiptera, Nematoda and other groups^1^. It is the most widely produced microbial insecticide in the world, and has been used extensively in pest management in agriculture, forestry and public health primarily because of its safety to humans, livestock and the environment^2^,^3^. However, the spores and ICPs of Bt are susceptible to degradation when exposed to ultraviolet radiation in sun light, with water and other substances in the cell ionized. It results in shortening its persistence which reduces its activity and therefore limits its use as a pesticide^4^,^5^,^6^,^7^.

One of the main ways to improve the resistance of Bt to UV radiation is to improve the formulation by adding protective agents that absorb UV. Commercial preparations of Bt comes in various forms such as solids, liquids, powders, tablets, granules, ball agents, liquid suspensions, and oil emulsions^8^,^9^,^10^. Bt is a microbial insecticide and contains both living cells and crystal proteins. Ultraviolet degradation of Bt adversely affects its biological activity. There are several formulations that provide some protection but much more needs to be done. Improving the formulation to provide better protection from UV has been the bottleneck in improving its activity as a pesticide. The adjuvant used is quite varied such as a fluorescent whitening agent, Congo red, folic acid, molasses, lignin, cellulose, alginate, shellac, yeast, p-aminobenzoic acid, etc and they can enhance the biological activity as much as 1000 times and protect Bt from ultraviolet irradiation^11^,^12^,^13^,^14^,^15^,^16^,^17^. Although these additives have protect against UV degradation they have some shortcomings: (1) the compatibility of the UV protection agents is poor, because they often inhibit microbial growth and spore germination; (2) the UV protectant is unstable, easily evaporates, degraded by the sun, easily washed away by the rain and absorbed by plant leaves, causing loss of protection; (3) many of the organic chemicals used as ultraviolet absorbents such as dyes and fluorescent agents are suspected carcinogens. Therefore, there is a need for the development of a more effective Bt formulation that is environmentally safe and has broad prospects of wide application.

Microencapsulation is a promising way to prepare Bt for use in the field, not only to resist ultraviolet radiation, but also to improve the slow release of the product as well as extend the period of the residual effect^10^. Sodium alginate is the most studied and most effective microencapsulation method. Sodium alginate solution containing the Bt is titered into the CaCl_2_ solution using different pore sized needles. The resulting microcapsule size depends on the size of the needle size and the viscosity of the sodium alginate solution^18^. This method has the advantage of simplicity, ease of operation, low cost and high embedding rate, and therefore has become the preparation method of choice for most commonly used Sodium alginate gel beads^19^,^20^,^21^. So, in order to improve the persistence of Bt product, we adopted the piercing-solidifying method of cell immobilization for the mosquitocidal Bt LLP29-M19, and the addition of hollow glass beads as a sustained release agent, natural melanin as a UV protectant. If a new formulation with higher mosquitocidal activity and longer persistence could be developed, it will be of great meaning not only for the knowledge of Bti, but also for the development of biological agents for mosquito management.

## Materials and Methods

### Bacterial strains and insects

Bt strain LLP29 used in our study was isolated from leaves of *Magnolia denudate*^22^. LLP29-M19 was obtained from mosquitocidal Bt LLP29 by repeated 19 times exposure to ultraviolet mutagenesis. *Culex quinquefasciatus* (Fafu strain), the mosquitoes used in bioassays, were collected from Fujian Agriculture and Forestry University, China and reared in our lab in a controlled environment room at 28 °C and 85% relative humidity (RH) with a photoperiod of 14:10 h (L:D)^3^.

### The basic medium and cell immobilization

Using the method of Prabakaran and Hoti^23^, we combined sodium alginate 2.5 g, LLP29-M19 bacteria powder 2 g, hollow glass beads 2 g, melanin black pigment 0.01 g, sodium benzoate 0.3 g and 0.5 g mouse feed in 100 ml distilled water. The mixed solution was added drop wise to a 4% CaCl_2_ solution using a 10 ml sterile syringe which resulted in bead formation. After solidifying in CaCl_2_ solution for 4 h, the granular beads were washed 2 times in distilled water and dried.

### Bioassay

**Figure 1:**
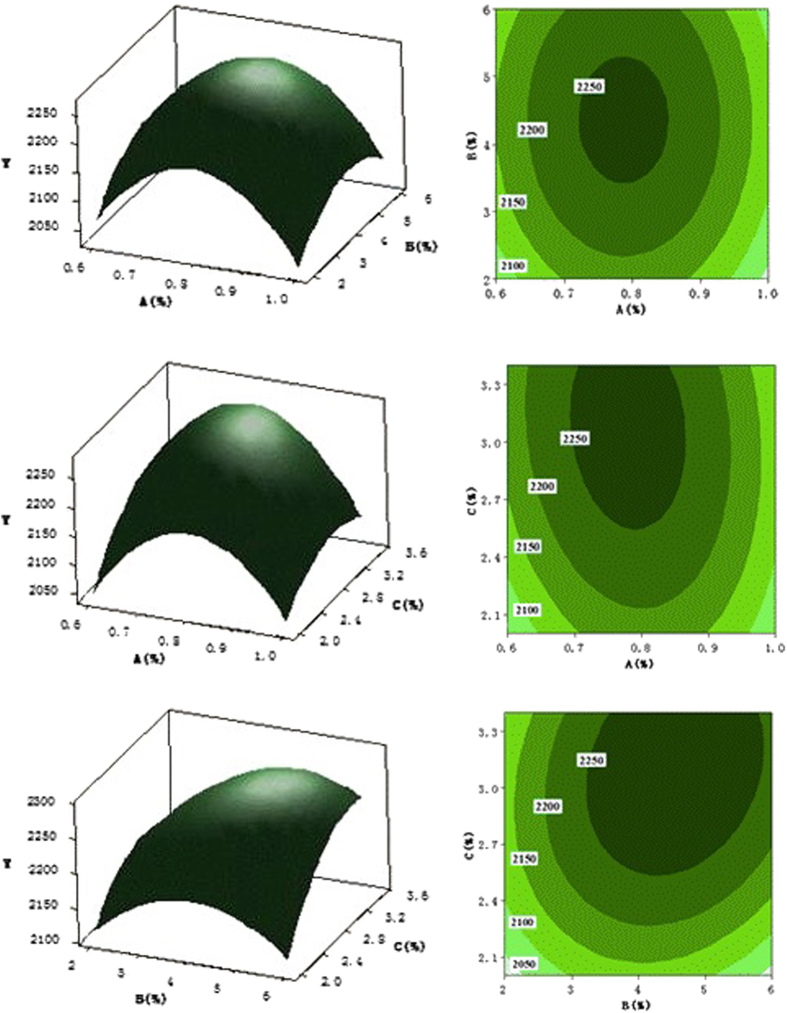
Three-dimensional graphs and contour patterns of the function. Y = f (**A,B**), Y = f (**A,C**), Y = f (**B,C**), A = sodium alginate (%), B = CaCl_2_ (%), C = hollow glass bead (%), Y = mosquitocidal activity.

Toxicity of the Bt formulation was tested on the third (penultimate) larval instar of *C. quinquefasciatus*. The samples were prepared in 0.3 g volumes containing 30 larvae each. Each sample was determined by comparing the final mortality with that of an untreated control. The mortality of larvae was statistically analyzed counting the number (N) every 24 h (until the N reached zero). Dechlorinated water and mosquitoes were changed every 24 h. Each experiment was repeated 3 times. The corrected mortality (marked as n) indicated the mosquitocidal effect and the total area of the 3-dimentional graphs indicates the mortality curve and the horizontal axis was representation of the sustained-release formulation to kill mosquito virulence effect (Fig. 1).


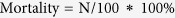



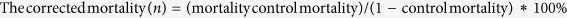


the mosquito killing virulence effect of the sustained-release formulation = ∑n − n_1_/2 (the n_1_ was representation of the first day of the corrected mortality, the n was representation of the last day of the corrected mortality)^24^,^25^. Meanwhile, toxicity of sample preparations was determined and compared as LT50 value^26^.

### Experimental design of the optimization of the sustained-release formulation

Single factor test using each component was conducted according to the references with some revision^27^.

1. Effect of sodium alginate on slow-release of mosquitocidal activity.Keeping all other conditions of the mixture unchanged, the effect sodium alginate concentrations of 0.5%, 1%, 1.5%, 2% and 2.5% was tested. After immobilization, the mosquitocidal toxicity of the controlled-release agent was determined, and the sustained-release mosquitocidal toxicity was calculated^27^.
2. Effect of CaCl_2_ on mosquito toxicity of the slow-release formulation was tested.After determining the optimal concentration of sodium alginate, CaCl_2_ concentrations of 2%, 3%, 4%, 5% and 6% were tested, keeping all other conditions unchanged. After immobilization, the mosquitocidal toxicity of the controlled-release agent was determined, and the sustained-release mosquitocidal toxicity was calculated^27^.
3. Effect of hollow glass bead on slow-release mosquitocidal toxicity.

After the optimal concentrations of sodium alginate and CaCl_2_ were determined, the hollow glass bead concentrations of 1.5%, 2%, 2.5%, 3% and 3.5% were tested, keeping all other conditions unchanged. After immobilization, the mosquitocidal toxicity of the controlled-release agent was determined, and the sustained-release mosquitocidal toxicity was calculated^27^.

### Screening of significant variables using PB design

The PB experimental design was applied to screen significant variables that influence the release of Bt. Eight variables of medium composition and culture conditions were tested at high (+1) and low (−1) levels based on a PB matrix design, which is a fraction of a two-level factorial design (Table 1)^28^,^29^.

### Path of steepest ascent

The initial estimates of operating conditions for the system were experimentally verified for accuracy using the method of steepest ascent to arrive at a system that provides maximum increase in response^28^.

The path of steepest ascent was carried out according to the coefficients and characters in the PB design, and the concentrations of the main variables in the formulation were adjusted to provide the best mosquito larvicidal activity. The path of steepest ascent is the line through the center of the region of interest and is normal to the fitted surface contours.

### Optimization of response surface analysis

The results from the PB design, steepest ascent and Box-Behnman analysis were collectively evaluated using MINITAB 16.0 software to come up with a response surface analysis.

## Results

### Effect of sodium alginate concentration on mosquito larvicidal activity

In order to determine the mosquito larvicidal activity of the controlled-release formulation, several concentrations of sodium alginate were tested, 0.5%, 1.0%, 1.5%, 2.0% and 2.5%. The highest toxicity was observed with 0.5% sodium alginate, slightly less with 1.0%, and even less with 1.5%. Because the formability of the formulation is not good with 0.5%, 1.0% sodium alginate was selected for further study.

### Effect of CaCl_2_ concentration mosquitocidal activity

Using a sodium alginate concentration of 1.0%, five different concentration of CaCl_2_ was evaluated. Bioassays indicated that the toxicity was the highest at a CaCl_2_ concentration of 2.0%, lower at 4.0%, and still lower at 3.0%. A CaCl_2_ concentration of 2.0% was therefore selected as the best immobilization cross-linker.

### Effect of hollow glass bead concentration on mosquitocidal activity

Using 1.0% of sodium alginate and 2.0% of CaCl_2_, different concentrations of hollow glass beads were compared. It was found that the toxicity was the highest when the concentration of hollow glass beads was 2.0%, and decreased in the order of 3.5% and 2.5%. Even though the highest toxicity was observed at a hollow glass bead concentration of 2.0%, since the suspension was not good at that concentration, a concentration of 3.5% was selected for the design of PB.

### PB design

According to single factorial experiments, the most suitable concentrations of sodium alginate, CaCl_2_ and hollow glass bead were 1.0%, 2.0% and 3.5%, respectively. Then, 8 factors, 2 variables and 12 replicates were evaluated using a PB design. Eight factors were considered in the formula: concentrations of sodium alginate (A), CaCl_2_ (B), hollow glass beads (C), bacteria (D), melanin (E), sodium benzoate (F), and mouse feed (G) as well as immobilization time (H).

In order to estimate the experimental error and check the adequacy of the first-order model, eight added to the variables of real interest. The PB test results were analyzed using MINITAB16.0 software (Table 1), and acquire Y (correction release mosquitocidal toxicity) with multivariate regression equation model of a:

Y = 1660.830–346.389 A + 363.611B-187.499 C + 102.221D + 46.944E-93.889F-45.001G-5.278 H. In addition, the model of R^2^ = 98.28%, show that the test data of the model change in 1.72% and the change of test data can be used to explain the 98.28% model, high reliability.

As shown in Table 2, the credibility level was greater than 90% for CaCl_2_ (P = 0.003), sodium alginate (P = 0.004), hollow glass beads (P = 0.022) and bacteria (P = 0.096). Their importance release of formulations of mosquitocidal virulence order as follow: CaCl_2_ > sodium alginate > hollow glass micro bead > bacteria. However, the melanin, sodium benzoate, mouse feed and other factors are fixed time on dosage form larvicidal activity had no significant effect.

The PB design revealed that in the sustained-release formulation CaCl_2_, sodium alginate and hollow glass beads were the 3 prime factors of the 8 factors affecting the release effect. The path of steepest ascent experiment was adopted to approach the optimal region of the formulation composition. The optimal combined concentration and mutual effect of the three factors were then optimized by response surface methodology (RSM). The result showed that the optimum formulation composition was sodium alginate 0.78%, CaCl_2_ 4.52%, hollow glass beads 3.12%, bacterial powder 3.0%, melanin 0.015%, sodium benzoate 0.2%, mice feed 0.5%, immobilization time 4.5 h, at which the corrected sustained-release virulence increased up to 6.07-fold higher than that of the basic formulation. This result differed from that forecast by RSM by only 5.0%.

### Steepest ascent design

The PB test determined that the factors with the most significant influence on virulence of the sustained-release mosquitocidal formulation were the concentrations of CaCl_2_, sodium alginate and hollow glass beads. According to a fitting equation of the steepest ascent method PB experiments, the optimal concentration range of the test arrangement and data were obtained (Table 3). Results are displayed in the x + 4ΔX (ΔX refers to the increment coefficient) that at concentrations of 0.8% sodium alginate, 4% CaCl_2_ and 2.7% hollow glass micro beads, the corrected mosquitocidal toxicity of the sustained-release formulation reached the highest point (2481.67). Therefore, we chose those concentrations as the center point of the follow-up response surface optimization test.

### Optimization of response surface analysis

#### Establishment of the model and analysis of two response surface regressions

According to the central composite design principle of the Box-Behnken, the 3 most important influence factors with 3 levels each are designed, with the levels of −1, 0, and 1. Response surface and the design of 3 factors and 3 levels, in total 15 test points of test analysis were carried out. It was found that A, B and C corresponded to sodium alginate (0.6, 0.8 and 1.0), CaCl_2_ (2.0, 4.0 and 6.0) and hollow glass beads (2.0, 2.7 and 3.4), respectively (Table 4).

The data were analyzed by MINITAB16.0 software and the model of regression quadratic response surface was established; obtained the regression polynomial fitting equation was as follows:

Y = 2261.111111–15.916667*A + 22.291667*B + 41.958333*C-127.430556*A*A−1.833333*A*B-19.166667*A* C-62.180556*B*B + 20.250000*B*C -41.180556*C*C.

The coefficient of determination of the quadratic response surface regression model, R^2^ = 90.59%, shows that the three factors model and two items can explain the dosage sustained-release mosquitocidal Virulence Variation of 90.59%, a good fitting degree. Sustained release mosquitocidal toxicity regression coefficient correction results show that C (P = 0.045), A*A (P = 0.003), B*B (P = 0.044) are significant. The coefficient of C (41.958333 > 0) is positive, and has a positive effect on the release of mosquitocidal toxicity; A*A (−127.430556 < 0) and B*B (−62.180556 < 0) have a negative effect on the release of mosquitocidal toxicity (Table 5). Analysis of variance shows that the general regression model P = 0.04, the reliability is high and the regression model is significant, so the model can be used for theoretical analysis and predictive formulation sustained-release mosquitocidal virulence optimization. At the same time, a square term in the model of P = 0.01 is also significant, while the model lack of fit term P value = 0.217 is not significant (Table 6).

In order to show the effects of concentrations of sodium alginate, CaCl_2_ and hollow glass beads on the slow release effect and characterization of the response surface character more directly, we used MINITAB16.0 to make the following two variables for the coordinates of the 3D map. By keeping one factor at the optimal condition and the relationship between the other two factors and response value with the response surface of 3D graph representation, we can directly reflect the effect of each factor on the response value relationship (Fig. 1).

#### Determination of the most suitable concentration of sustained-release formulations

The use of MINITAB16.0 software the optimizer divides model of three group analysis received maximum correction sustained-release mosquitocidal virulence formulation for optimal concentration, A = 0.78%; C = 3.12%, B = 4.52%; prediction correction release mosquitocidal toxicity was 2277.74. The best sustained-release mosquitocidal virulence formula composition was (%): sodium alginate 0.78 and CaCl_2_ 4.52, hollow glass bead 3.12, bacteria 3, melanin 0.015, sodium benzoate 0.2, mice feed 0.5 and an immobilization time of 4.5 h.

#### Verification of the optimized model

By the model get the optimal release agent and the immobilized test was carried out, the test results the release mosquitocidal toxicity was 2391.67, and the model predicted value is only a difference of 5% (2277.74), which shows that the model can better predict actual immobilization and also proves that the optimization model is effective.

Only from the view of toxicity in the limited time, the optimized formulation is similar to Bti LLP29 wettable powder and 5100 ITU/mg standards IPS82 as reported in our previous work, whose LC50 were 1.487 ITU/mg and 0.35 ITU/mg, respectively^25^. In order to value the toxicity and persistence in the field, LT50 of the new formulation was also detected. Results found that LT_50_ of the optimized new formulation is 23.707 (22.339, 25.187) d, great higher than that of the control 5.069 (3.457, 6.298) d.

## Discussion

After more than 70 years of chemical pesticide use, biological control products are increasingly used in modern agriculture. However, because they are very expensive and often not reliable, the market of bio-pesticides is still limiting^30^. It is similar with Bt. Since 1930 s, Bt has been focused on interest of insect control. It has been materialized by first usage in 1933, and then been used as pesticide in agriculture, forestry and mosquito control since the 1950 s^30^. However, Bt insecticide is also adversely affected by the environmental factors in a variety of ways when used in the field, which leads to low toxicity and reduced persistence. It is even be inactive quickly when exposed to UV and ionizing radiation, with the proteins cross-linked by hydroxyl radicals and ionizing radiation^31^,^32^, especially the UV-B rage (280–330 nm) of the solar spectrum reaching Earth’s surface.

Formulation technologies have been used to improve the number of commercial biological control products, and various compounds were added to active agents, such as formulations helping with long activity persistence^33^,^34^,^35^. Research on adding sustained-release capsules and natural UV protection, such as melanin and pigment, not only has a stabilizing effect on the insecticide, but also has a very good protective function against adverse environmental factors such as ultraviolet radiation, and can also greatly prolong the persistence of efficacy of Bt formulations^30^,^31^,^34^,^36^.

So, there are many factors considered in this study to improve the resistance of spores and toxins to environmental stresses including sunlight. Sensitivity of Bti spores to UV-B radiation depended upon their culture age. 48 h of culture was thought to be the maximal resistance to UV-B, and 24 h approached its maximal larvicidal activity^37^. In our previous study, a highly resistant Bt mutant, named LLP29-M19, was obtained after UV repeated exposure (Data has not been published). It was firstly considered for new Bt formulation research that provides superior protection against UV and permits sustained release against mosquito larvae. Melanin is a natural pigment that is easily biodegradable in the natural. It is reported to absorb radiation consequently photo protection of Bt^37^. Therefore, melanin was secondly considered to appear in the new formulation for prolonging the toxicity of Bt product under field conditions and UV irradiation. Larvae of mosquitoes live in the water. Water environment is quite different from terrestrial typical for majority of insects, such as Lepidoptera, Coleoptera, Hemiptera, etc. From this point of view formulation of Bti requires special attention. In many shortages of Bti, deposition of Bti product is an important one to limit the mosquitoes’ eating and make the product less efficacy. So, floatation improvement of the new formulation is another main target in this study. Then the piercing-solidifying method of cell immobilization and the addition of hollow glass beads as a sustained release agent were used.

In this study, the new formulation with higher mosquitocidal activity and longer persistence has thus been developed using the screening method of Plackett-Burman (PB) test and the optimization design of sustained-release formulation using the surface response test of Box-Behnken. The optimum sustained-release formulation obtained by response surface methodology was 0.78% sodium alginate, 4.52% CaCl_2_, 3.12% hollow glass beads, 3% LLP29-M19 bacteria powder, 0.015% melanin, 0.2% sodium benzoate, 0.5% mice feed and an immobilization time of 4.5 h. When the optimal formulation of sustained-release agent concentration was used in bioassays, the results showed that the corrected mosquitocidal virulence reached 2391.67. This was within 5% of the predicted value, and illustrates the response optimization experiment method can be successfully applied to the formulation of the surface analysis. The toxicity of the optimized formulation of the slow-release mosquitocide was 6.07 times that of the basic formula, which is similar to the results of LT_50_, and the new formulation might provide broad application prospects. It will potentially help in the design of better strategies or formulations with limited spraying, and do some good to solve the problem of Bt’s susceptibly to UV and being with longer survival under field condition.

## Additional Information

**How to cite this article**: Zhang, L. *et al*. A new formulation of Bacillus thuringiensis: UV protection and sustained release mosquito larvae studies. *Sci. Rep.* **6**, 39425; doi: 10.1038/srep39425 (2016).

**Publisher's note:** Springer Nature remains neutral with regard to jurisdictional claims in published maps and institutional affiliations.

## Figures and Tables

**Table 1:** PB experimental design and results for screening of culture conditions affecting the mosquitocidal toxicity of the release formulation.

Run	A	B	C	D	E	F	G	H	mosquitocidal activity
1	−1	1	−1	−1	−1	1	1	1	2206.67 ± 28.28
2	−1	1	1	−1	1	−1	−1	−1	2216.67 ± 47.14
3	1	1	−1	1	−1	−1	−1	1	1968.33 ± 16.50
4	1	−1	1	−1	−1	−1	1	1	746.67 ± 18.86
5	−1	−1	−1	−1	−1	−1	−1	−1	1881.67 ± 11.79
6	−1	−1	−1	1	1	1	−1	1	2015.00 ± 40.07
7	1	1	1	−1	1	1	−1	1	1436.67 ± 51.85
8	1	1	−1	1	1	−1	1	−1	2155.00 ± 58.93
9	−1	1	1	1	−1	1	1	−1	2163.33 ± 84.85
10	1	−1	1	1	−1	1	−1	−1	716.67 ± 28.28
11	−1	−1	1	1	1	−1	1	1	1560.00 ± 75.42
12	1	−1	−1	−1	1	1	1	−1	863.33 ± 47.14

**Table 2:** Analysis of PB results.

	effect	Coefficient	Standard error	T value	P value	Important sequence
Intercept	—	1660.830	42.63	38.96	0.000	—
A	−692.8	−346.389	42.63	−8.13	0.004	2
B	727.2	363.611	42.63	8.53	0.003	1
C	−375.0	−187.499	42.63	−4.40	0.022	3
D	204.4	102.221	42.63	2.40	0.096	4
E	93.9	46.944	42.63	1.10	0.351	6
F	−187.8	−93.889	42.63	−2.20	0.115	5
G	−90.0	−45.001	42.63	−1.06	0.369	7
H	−10.6	−5.278	42.63	−0.12	0.909	8

**Table 3:** Design and data from the steepest ascent experiment.

Order	Sodium alginate (%)	CaCl_2_ (%)	Hollow glass bead (%)	Mosquitocidal activity
x	1.00	2.0	3.5	1643.33 ± 14.14
x + 1△x	0.95	2.5	3.3	1711.67 ± 40.07
x + 2△X	0.90	3.0	3.1	1850.00 ± 37.71
x + 3△X	0.85	3.5	2.9	2338.33 ± 63.64
x + 4△X	0.80	4.0	2.7	2481.67 ± 25.93
x + 5△X	0.75	4.5	2.5	1405.00 ± 44.78
x + 6△X	0.70	5.0	2.3	745.00 ± 30.64
x + 7△X	0.65	5.5	2.1	441.67 ± 2.36

**Table 4:** Design and data from the response surface methodology.

Orders	A	B	C	Mosquitocidal activity
1	0	0	0	2281.67 ± 77.78
2	−1	1	0	2098.33 ± 73.07
3	1	−1	0	2048.33 ± 51.21
4	−1	−1	0	2060.00 ± 56.57
5	0	0	0	2230.00 ± 88.28
6	0	1	−1	2091.67 ± 46.50
7	1	0	−1	2076.67 ± 61.28
8	−1	0	−1	2086.67 ± 51.85
9	−1	0	1	2146.67 ± 69.43
10	1	1	0	2079.33 ± 57.07
11	1	0	1	2060.00 ± 44.71
12	0	−1	1	2183.33 ± 78.28
13	0	−1	−1	2077.67 ± 66.00
14	0	0	0	2271.67 ± 58.93
15	0	1	1	2278.33 ± 77.78

**Table 5:** Estimated value of coefficient in the regression equation.

	Coefficient	Standard error	T value	P value
Intercept	2261.111111	25.81	87.602	0.000
A	−15.916667	15.81	−1.007	0.360
B	22.291667	15.81	1.410	0.218
C	41.958333	15.81	2.655	0.045
A*A	−127.430556	23.27	−5.477	0.003
B*B	−62.180556	23.27	−2.673	0.044
C*C	−41.180556	23.27	−1.770	0.137
A*B	−1.833333	22.35	−0.082	0.938
A*C	−19.166667	22.35	−0.857	0.430
B*C	20.250000	22.35	0.906	0.407

**Table 6:** Anova analysis of model equations.

	DF	Seq SS	Adj SS	Adj MS	F value	P value
Regression	9	96209	96208.5	10689.8	5.35	0.04
Linear	3	20086	20086.1	6695.4	3.35	0.113
Quadratic	3	72999	72999.3	24333.1	12.17	0.01
Cross product	3	3123	3123.1	1041	0.52	0.686
Residual error	5	9993	9993.2	1998.6	—	—
Lack of fit	3	8491	8491.3	2830.4	3.77	0.217
Pure error	2	1502	1501.9	750.9	—	—
Total error	14	106202	—	—	—	—
